# Nationwide trophic cascades: changes in avian community structure driven by ungulates

**DOI:** 10.1038/srep15601

**Published:** 2015-10-26

**Authors:** Georgina Palmer, Philip A. Stephens, Alastair I. Ward, Stephen G. Willis

**Affiliations:** 1 School of Biological and Biomedical Sciences, Durham University, South Road, Durham, DH1 3LE; 2 Department of Biology, University of York, York, YO10 5DD; 3 National Wildlife Management Centre, Animal and Plant Health Agency, York YO41 1LZ; 4 School of Biological, Biomedical and Environmental Science, University of Hull, Cottingham Road, Hull, HU6 7RX.

## Abstract

In recent decades, many ungulate populations have changed dramatically in abundance, resulting in cascading effects across ecosystems. However, studies of such effects are often limited in their spatial and temporal scope. Here, we contrast multi-species composite population trends of deer-sensitive and deer-tolerant woodland birds at a national scale, across Britain. We highlight the divergent fates of these two groups between 1994 and 2011, and show a striking association between the calculated divergence and a composite population trend of woodland deer. Our results demonstrate the link between changes in deer populations and changes in bird communities. In a period when composite population trends for deer increased by 46%, the community population trend across deer-sensitive birds (those dependent on understory vegetation) declined much more than the community trend for deer-tolerant birds. Our findings suggest that ongoing changes in the populations of herbivorous ungulates in many countries worldwide may help explain patterns of community restructuring at other trophic levels. Ungulate impacts on other taxa may require more consideration by conservation practitioners than they currently receive.

Herbivorous ungulates exert cascading effects on components of biodiversity in ecosystems they inhabit, including birds, small mammals, meso-herbivores and invertebrates^1^,^2^,^3^,^4^. These effects are commonly mediated through changes to vegetation abundance, structure, and diversity^1^. Given these effects, there is a pressing need to understand the potential consequences of on-going changes – both increases and declines – in the populations of herbivorous mammals^1^,^4^,^5^,^6^,^7^,^8^, such as deer. Although the most commonly studied trophic cascades are of predator-consumer-producer^9^, these cascades can, and have, been extended to include the indirect effect of the consumer on other species. This is exemplified by the wolf-elk-tree system in Yellowstone National Park, USA; Ripple & Beschta^2^ demonstrated that the restoration of riparian habitats as a result of increased predation of consumers (elk, *Cervus elaphus*) by predators (wolves, *Canis lupus*), resulted in further cascades to beavers *Caster Canadensis* and bison *Bison bison*. Here, we investigate an apex consumer (deer)-producer (plant)-consumer (bird) cascade, in a system without apex predators.

Increasing browsing pressure from deer has been proposed to be one of the key contributors to the recent, rapid declines of temperate woodland birds^10^,^11^,^12^. Indeed, local-scale experimental studies have shown relationships between an increase in deer abundance and a decrease in the abundance or diversity of birds^1^,^12^. For example, roe *Capreolus capreolus*, fallow *Dama dama* and muntjac *Muntiacus reevesi* deer have been shown to affect the abundance and diversity of shrub layer plants, resulting in cascading impacts on several bird species^13^,^14^.

In countries where extensive long-term avian monitoring schemes exist, composite population trends of birds have been used to demonstrate community-level changes in the abundance of woodland birds^10^,^12^. Composite trends are used as they balance the magnitude and number of declining and increasing trends, and therefore provide a measure of average change in that community^15^. Community-level indices have also been used to create generalised indicators of environmental impacts on animal populations. For example, Gregory *et al.*^16^ created a ‘Climate Impact Indicator’, which measured the divergence of population trends of birds in two groups: those expected to be favourably, or adversely, affected by climate change. Such indicators are easy to interpret and highly useful for describing general patterns of change in impacts over time, raising awareness of the environmental driver, and assisting in setting strategies to reduce negative impacts^16^.

Despite evidence that high deer densities can result in negative effects on some bird species^17^,^18^, temporal trends in avian community abundance have not been directly related to temporal trends in ungulate abundance. Here, we develop a generalised indicator of the impacts of deer on woodland bird communities, which we term the ‘Deer Impact Indicator’ (DII). Our indicator is based on a long-running, randomised and high resolution dataset of both bird and mammal abundances, collected at a national scale across Britain between 1994 and 2011. We explore the influences of both deer and climate on the DII.

## Results

**Figure 1:**
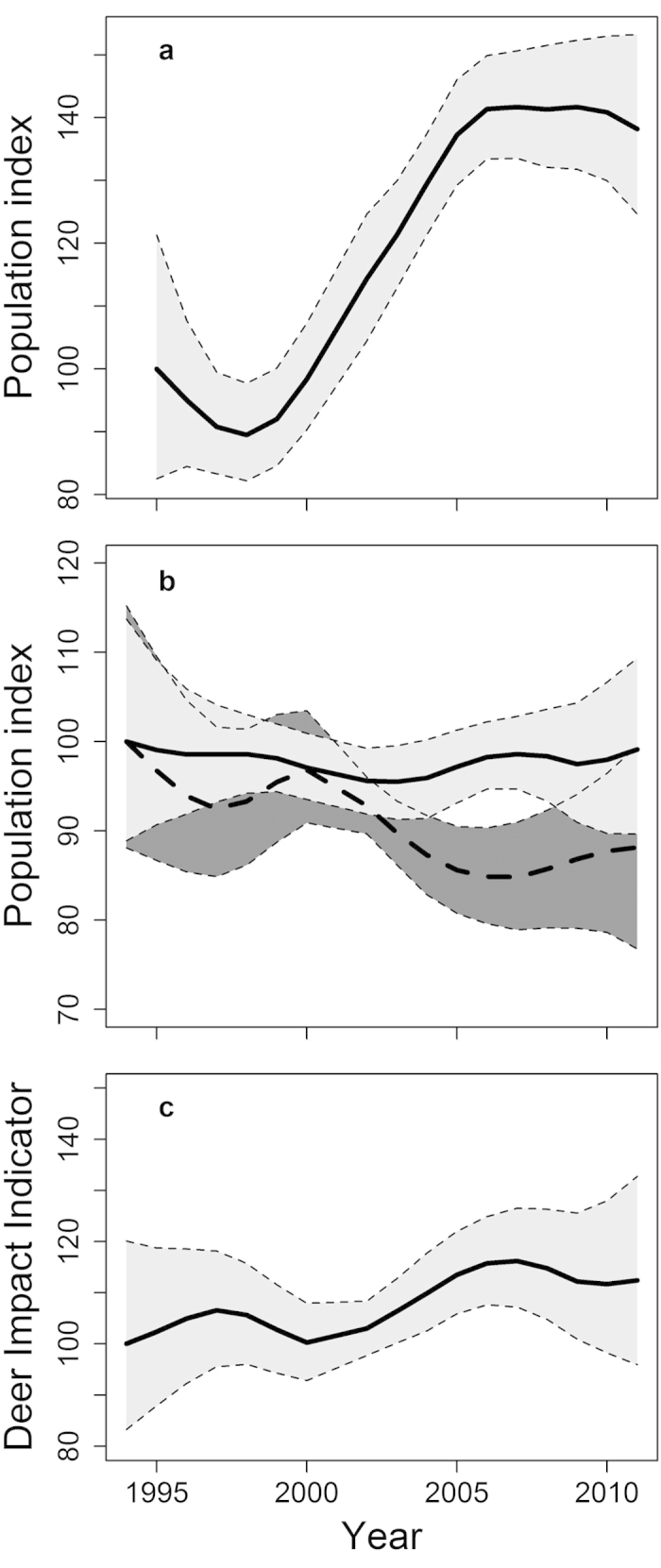
Composite population trends (panels (a,b)) and the Deer Impact Indicator (DII, panel (c)). In panels (**a,b**), the bold lines represent the composite index for deer (**a**), deer-sensitive birds (n = 15, bold dashed line on panel (**b**)) and deer-tolerant birds (n = 19, bold solid dashed line on panel (**b**)); any value above 100 represents an increase in the index relative to the start year, and vice versa. In panel (**c**), the solid bold line represents the DII, which is the ratio of the composite population index for deer-tolerant birds to that of deer-sensitive birds. Shaded polygons around each bold line represent the 90% bootstrap confidence intervals for annual values, from 10,000 bootstrapped replicates (Materials and Methods; Gregory, et al.^16^).

The composite population trend for deer increased by 46% between 1996 and 2010 (Fig. 1a). Individual population trends of birds were not unidirectional; twelve (63%) of the deer-tolerant bird species increased between 1995 and 2010, while seven (37%) declined (Supplementary Table S1). Conversely, seven (47%) of the deer-sensitive species increased over the same time period, and eight (53%) decreased (Supplementary Table S1). However, we found that populations of deer-sensitive bird species, considered together, declined by 9% between 1995 and 2010, while populations of deer-tolerant bird species declined by only 1% over the same time period (Fig. 1b).

The DII, which contrasts the fates of deer-sensitive and deer-tolerant bird species, increased by 9% between 1995 and 2010 (Fig. 1c) and showed a very strong, significant positive relationship with the composite deer trend, after accounting for a one-year lag (see Methods, *β* = 0.23, χ^2^ (1,11) = 296.80, *p* < 0.0001). The most parsimonious model explained 86% of variation in the DII trend; there was limited support for a relationship between the DII and climate (Supplementary Table S2).

## Discussion

Recent increases in deer populations across Britain were associated with greater divergence in the composite population trends of woodland bird communities tolerant of, and sensitive to, deer. We found that variations in the DII were significantly related to preceding changes in deer abundance; on average, the deer-sensitive bird species suffered a decline between 2000 and 2007 (Fig. 1b), and the increase in deer abundance since 1998 (Fig. 1a) suggests a link between these two trends. Additionally, since 2006 deer abundances in our study sites appear to have stabilised (Fig. 1a); the time lag between changes in deer population trend and in the DII is illustrated by the stabilisation of the DII since c.2007 (Fig. 1c). These findings indicate that deer have a delayed impact on understorey bird populations^10^, through indirect impacts on shrub layer vegetation as a result of herbivory^1^,^19^. Removal of understorey vegetation by deer may negatively impact birds directly through the loss of nesting and foraging habitats, but may also increase the risk of nest predation^20^. Conversely, however, some bird species such as the tree pipit *Anthus trivialis*, wood warbler *Phylloscopus sibilatrix* and redstart *Phoenicurus phoenicurus*, may benefit from the presence of deer, either because they require the more open structures created by deer, or as a result of reduced competition with other birds^14^,^21^,^22^. As such, management to stabilise or reverse the trend of declining deer-sensitive woodland birds – such as by increasing woodland planting or by erecting deer fences – may have the simultaneous, negative effect of reducing abundances of species which rely on open woodland habitats.

Observed intra-group differences in individual population trends suggest that the choice of birds included in the DII calculations might have a bearing on the DII. Such intra-group differences are to be expected, as cascading effects are complicated and do not impact on all species in the same way. However, four factors provide reassurance that the choice of species to include would not change our overall conclusions. First, we included in the index only those species independently classified as ‘woodland birds’^23^ (Supplementary Table S3), as we expected deer to have a pronounced effect on woodland species^14^. Second, we grouped birds into deer-tolerant and deer-sensitive in line with previous studies^10^,^14^. Third, bootstrapped confidence intervals around the DII suggest that the index is robust to variation in the chosen species. Fourth, while there was variation in individual population trends within our two bird groups, this is both unsurprising and relatively unimportant to our broader conclusions. In particular, it is likely that competition occurs between bird species within each of the groups, potentially resulting in trends for some that are counter to expectation. As a result, it is important to focus on the overall fate of each group, rather than on single-species trends.

Although we cannot attribute causality with confidence based on these observational data, our focus on the contrasting fates of two sympatric groups of species controls for many potential confounding processes. While other, unmeasured factors may have influenced changes in bird abundance over time, our temporally and spatially extensive analyses are consistent with more intensive, local-scale studies of deer impacts on deer-sensitive birds^13^,^18^,^19^. Unmeasured factors that influence woodland bird populations include changes in the age structure and/or management of woodlands^10^ and in neighbouring habitats^11^. However, we found limited evidence for climatic influences on the DII (Supplementary Table S2) and, unfortunately, there is a lack of available data to ascertain whether land-use or land-management changes have affected species’ abundances. Producing an index based *a-priori* on species vulnerable and tolerant to deer and then demonstrating consistent divergence in their population trends is a compelling argument for deer being a major contributor to these recent trends. Although multiple drivers – not just deer – have been influencing birds, the strong correlation between the DII and deer abundance is striking. Confidence in our conclusions would be increased by analyses showing that regional DIIs were consistent in magnitude with relative changes in deer abundance in those areas. Unfortunately, the data on deer abundance – based on opportunistic sightings during bird surveys – are too noisy for analyses at a smaller spatial scale.

We have shown that recent changes in the abundance of woodland birds are strongly associated with preceding changes in the abundance of British deer. These results are consistent with the rapid, landscape-scale cascading impacts herbivores can have on avian community structure^3^. While some large herbivore species, especially deer, are expected to continue to increase in the future (e.g. Irvine *et al.* (2007) in Ref. 14 and 24), many other large herbivores are declining and are now listed as threatened with extinction^4^. Under both circumstances, significant ecological changes to vegetation and cascading impacts on the species that depend on these habitats seem likely^25^. Land managers can therefore either accept the dynamic nature of systems under their care, or adapt their management strategies in light of the likely impacts on species of conservation, economic, ecological or cultural importance.

## Material and Methods

### Data sources

We use count data collected as part of the British Trust for Ornithology’s Breeding Bird Survey (BBS; which also records mammal species) monitoring scheme. We use data from 1811 predominantly woodland BBS sites in our analyses, excluding sites with more than 25% urban or upland habitat, as well as those with less than 20% woodland cover (from Land Cover Map 2000 data; www.ceh.ac.uk/AccessingLCMData.html; date of access 27/03/2015). BBS data for 2001 were excluded from all analyses due to a reduction in recording effort in that year, the result of a national foot-and-mouth disease outbreak which limited access to the countryside^26^,^27^. We considered the impact of the three most widespread and abundant British deer species – fallow deer, roe deer and Chinese muntjac deer – on woodland birds. Red deer *Cervus elaphus*, Chinese water deer *Hydropotes inermis*, and sika deer *Cervus nippon* are poorly monitored by the BBS^14^, so were excluded from analyses. The BBS deer count data have been shown to correlate well with deer density estimates collected using more labour-intensive methods^14^.

Species classed as ‘woodland birds’ by the Department for Environment, Food and Rural Affairs (DEFRA)^23^, were split into two groups (those considered to be ‘deer-tolerant’ [*n* = 19], and those negatively affected by deer herbivory, hereafter termed ‘deer-sensitive’ [*n = *15]) based upon their dependence on understorey vegetation for feeding and/or nesting habitat (Supplementary Table S3). There were insufficient data to calculate a population trend for the hawfinch *Coccothraustes coccothraustes.*

Annual climate data from 1995 to 2011 were extracted at a 5 km^2^ resolution from the UK Met Office (www.metoffice.gov.uk/climatechange/science/monitoring/ukcp09; date of access 27/03/2015). For the 5 km cells associated with the 1811 BBS sites, we calculated the mean growing degree days above 5 °C (GDD5) and mean temperature of the coldest month (MTCO) each year, across all BBS sites.

### Species-specific population trends

Population trends for each bird and deer species were obtained by fitting generalised linear models to count data (obtained from the BBS) using a log link function, assuming a poisson- (roe deer, muntjac deer, and birds) or negative binomial- (fallow deer, to reduce the influence of herding behaviour of this species, following Newson *et al.*^26^) error distribution and accounting for over-dispersion^28^. Smoothed population trends for each species were then calculated by fitting generalised additive models (GAMs) to annual indices, using a smoothed year effect with 5 degrees of freedom^29^. Smoothed species-specific population trends were calculated this way due to the difficulty of fitting GAMs directly to the bird census data (www.bto.org/about-birds/birdtrends/2011/methods/statistical-methods-alerts, date of access 27/03/2015). Smooth trends were used as they reduce (or remove, depending on the number of degrees of freedom) between-year fluctuations in population sizes, while retaining the major features of the trend^10^. We set the initial value of the trend to 100 in 1994 (1995 for deer), and then calculated annual trends for all years to 2011, relative to the population size in the reference year. When calculating changes in trend, the start and end years were truncated to ensure end effects (due to the use of GAMs) did not bias inference^29^.

### Composite population indices and the DII

For the deer-sensitive and deer-tolerant bird groups as well as for deer, we calculated composite population indices. Within each group, the composite index represents the geometric mean of each species’ relative abundance trend. The geometric mean was calculated by: first, taking the log of each species’ population trend (from 1994 to 2011 for birds and from 1995 – 2011 for deer); second, by calculating the arithmetic mean of those log-transformed values across all species in the group; and third, by taking the exponent of those values^30^. A geometric mean was used so that a doubling of the index from 100 to 200 was equivalent, but opposite, to a decline in index from 100 to 50.

90% confidence intervals for the composite deer population trend were calculated following two steps. Firstly, for each deer species in turn, sites were randomly re-sampled (with replacement) and annual trends re-calculated using GLM and GAMs (as above). The composite trend across each of the 10,000 bootstrapped replicates was then calculated.

For a given year, the DII was calculated as the ratio of the composite population index for deer-tolerant to that of deer-sensitive bird species. 90% bootstrap confidence intervals around the DII were calculated following four steps. First, bird species within each of the deer-tolerant and deer-sensitive groups were resampled with replacement; second, the resampled composite trend was then calculated (as above); and third, the DII was calculated. This process was repeated 10,000 times, and then 90% confidence intervals around the DII were obtained.

The influence of deer and of climate effects on the DII was assessed by fitting multi-predictor generalised linear models relating the DII to yearly GDD5, MTCO and composite deer trend values. We allowed for one year (climate) and up to three year (deer) time-lags in these relationships, but only allowed one deer term plus one term for each of GDD5 and MTCO, at most, given issues with colinearity between predictor variables. We included different possible lagged effects as we expected that changes in bird populations may be driven indirectly by deer-driven changes in habitat structure, and directly by changes in climate^10^. Candidate models were chosen as those within 6 AIC units of the most parsimonious model; to reduce the retention of overly-complex models, nested models were removed from the candidate model set following Richards *et al.*^31^.

## Additional Information

**How to cite this article**: Palmer, G. *et al.* Nationwide trophic cascades: changes in avian community structure driven by ungulates. *Sci. Rep.* **5**, 15601; doi: 10.1038/srep15601 (2015).

## Supplementary Material

Supplementary Information
