# Supplementary material for: Nationwide trophic cascades: changes in avian community structure driven by ungulates
Source: Sci Rep. 2015 Oct 26;5:15601. doi: 10.1038/srep15601 (PMC4620481; doi:10.1038/srep15601)
Supplement: Supplementary Information [file srep15601-s1.pdf]

**Supplementary Information**

# Nationwide trophic cascades: changes in avian community structure driven by ungulates

Georgina Palmer, Philip A. Stephens, Alastair I. Ward, and Stephen G. Willis

**Supplementary Table S1. Percentage change in population trends for woodland birds between 1995 and 2010.** When the 95% upper (UCL) and lower (LCL) confidence limits of the trend in 2010 do not overlap with 100, this indicates a significant change in population size for each species between the first and last years (marked with an asterisk (*) in the 'change' column).

| | Species | | | | | |
|---|---|---|---|---|---|---|
| Group | Common name | Scientific name | Change (%) | Sig | LCL | UCL |
| Deer-tolerant birds | Sparrowhawk | *Accipiter nisus* | -16 | * | 73 | 85 |
| | Long-tailed tit | *Aegithalos caudatus* | 10 | | 96 | 116 |
| | Tree pipit | *Anthus trivialis*^ | -8 | * | 75 | 95 |
| | Siskin | *Carduelis spinus* | 58 | * | 154 | 198 |
| | Treecreeper | *Certhia familiaris* | -9 | | 81 | 109 |
| | Greater spotted woodpecker | *Dendrocopos major* | 121 | * | 225 | 237 |
| | Lesser spotted woodpecker | *Dendrocopos minor* | -30 | * | 43 | 75 |
| | Chaffinch | *Fringilla coelebs* | 6 | * | 103 | 111 |
| | Jay | *Garrulus glandarius* | 2 | * | 86 | 98 |
| | Spotted flycatcher | *Muscicapa striata* | -44 | * | 39 | 55 |
| | Coal tit | *Parus ater* | 20 | * | 115 | 131 |
| | Blue tit | *Parus caeruleus* | 9 | * | 107 | 119 |
| | Great tit | *Parus major* | 36 | * | 132 | 144 |
| | Redstart | *Phoenicurus phoenicurus*^ | 4 | * | 104 | 132 |
| | Wood warbler | *Phylloscopus sibilatrix*^ | -56 | * | 11 | 31 |
| | Green woodpecker | *Picus viridis* | 43 | * | 135 | 155 |
| | Goldcrest | *Regulus regulus* | 6 | | 90 | 138 |
| | Nuthatch | *Sitta europaea* | 71 | * | 162 | 182 |
| | Tawny owl | *Strix aluco* | -26 | * | 53 | 81 |
| Deer-sensitive birds | Lesser redpoll | *Carduelis cabaret* | -12 | | 38 | 140 |
| | Robin | *Erithacus rubecula* | 9 | * | 103 | 115 |
| | Nightingale | *Luscinia megarhynchos* | -39 | * | 36 | 60 |
| | Willow tit | *Parus montanus* | -72 | * | 7 | 27 |
| | Marsh tit | *Parus palustris* | -25 | * | 62 | 82 |
| | Chiffchaff | *Phylloscopus collybita* | 56 | * | 138 | 178 |
| | Willow warbler | *Phylloscopus trochilus* | -24 | * | 76 | 84 |
| | Dunnock | *Prunella modularis* | 15 | * | 107 | 119 |
| | Bullfinch | *Pyrrhula pyrrhula* | -8 | * | 77 | 97 |
| | Blackcap | *Sylvia atricapilla* | 88 | * | 181 | 201 |
| | Garden warbler | *Sylvia borin* | -11 | * | 84 | 96 |
| | Lesser whitethroat | *Sylvia curruca* | 6 | | 81 | 101 |
| | Wren | *Troglodytes troglodytes* | -6 | * | 75 | 99 |
| | Blackbird | *Turdus merula* | 20 | * | 117 | 125 |
| | Song thrush | *Turdus philomelos* | 23 | * | 113 | 129 |

**Supplementary Table S2. Candidate models (≤ 6 AICc units from the best model) for describing variation in the Deer Impact Indicator.** Predictors were the composite deer trend from the current (t) and three previous years (t-1, t-2 and t-3), as well as (growing degree days above 5°C (GDD5) from the current and previous year. Models including mean temperature of the coldest month were > 6 AICc units from the best model, and so are not included in the table.

| Intercept | $Deer_t$ | $Deer_{t-1}$ | $Deer_{t-2}$ | $Deer_{t-3}$ | $GDD5_t$ | $GDD5_{t-1}$ | d.f. | logLik | Δ AICc |
|---|---|---|---|---|---|---|---|---|---|
| 81.89 | | 0.23 | | | | | 3.00 | -27.05 | 0.00 |
| 56.79 | | | 0.22 | | 0.01 | | 4.00 | -25.87 | 1.98 |

**Supplementary Table S3. Foraging preferences and nesting locations of woodland bird species**. Foraging and nesting locations are mainly taken from (A) Hewson & Noble[1], but are augmented and supported by: (B) Vanhinsbergh *et al.*[2], (C*) Newson *et al.*[3] and (D) Lewis *et al.*[4].

| | Species | | | | |
|---|---|---|---|---|---|
| Group | Common name | Scientific name | Foraging location | Nesting location | Refs |
| Deer-tolerant birds | Sparrowhawk | *Accipiter nisus* | Various | Tree | A |
| | Long-tailed tit | *Aegithalos caudatus* | Trees | Variable | A |
| | Tree pipit | *Anthus trivialis*^ | Ground | Ground | A |
| | Siskin | *Carduelis spinus* | Trees | Tree | B |
| | Treecreeper | *Certhia familiaris* | Trees | Cavity | A |
| | Greater spotted woodpecker | *Dendrocopos major* | Trees | Cavity | A |
| | Lesser spotted woodpecker | *Dendrocopos minor* | Trees | Cavity | A |
| | Chaffinch | *Fringilla coelebs* | Various | Tree | A; C |
| | Jay | *Garrulus glandarius* | Ground | Tree | A |
| | Spotted flycatcher | *Muscicapa striata* | Trees | Cavity | A |
| | Coal tit | *Parus ater* | Trees | Cavity | A |
| | Blue tit | *Parus caeruleus* | Trees | Cavity | A; C |
| | Great tit | *Parus major* | Various | Cavity | A |
| | Redstart | *Phoenicurus phoenicurus*^ | Ground | Cavity | A |
| | Wood warbler | *Phylloscopus sibilatrix*^ | Trees | Ground | A |
| | Green woodpecker | *Picus viridis* | Ground | Cavity | A |
| | Goldcrest | *Regulus regulus* | Trees | Tree | A |
| | Nuthatch | *Sitta europaea* | Trees | Cavity | A; C |
| | Tawny owl | *Strix aluco* | Ground | Cavity | A |
| Deer-sensitive birds | Lesser redpoll | *Carduelis cabaret* | Trees | Understorey | A |
| | Robin | *Erithacus rubecula* | Ground | Ground, rock crevice, tree roots | A; C* |
| | Nightingale | *Luscinia megarhynchos* | Ground | Understorey | A; B; C |
| | Willow tit | *Parus montanus* | Understorey | Cavity, shrub layer | A; B; C ,D |
| | Marsh tit | *Parus palustris* | Various; rarely away from cover | Cavity, tree roots, ground | A; B; C |
| | Chiffchaff | *Phylloscopus collybita* | Trees | Understorey | A; C |
| | Willow warbler | *Phylloscopus trochilus* | Understorey | Ground | A; B; C |
| | Dunnock | *Prunella modularis* | Ground | Understorey | A; B; C |
| | Bullfinch | *Pyrrhula pyrrhula* | Various | Understorey | A; B; C |
| | Blackcap | *Sylvia atricapilla* | Various | Understorey | A; C |
| | Garden warbler | *Sylvia borin* | Various | Understorey | A |
| | Lesser whitethroat | *Sylvia curruca* | Understorey | Understorey | A |
| | Wren | *Troglodytes troglodytes* | Understorey | Understorey | A |
| | Blackbird | *Turdus merula* | Ground | Understorey | A; C |
| | Song thrush | *Turdus philomelos* | Ground | Understorey | A; B; C |

* Newson *et al.*[3] class the robin *Erithacus rubecula* as a 'control species', not expected to be affected by deer. However, we include this species as a 'deer-sensitive' species given its feeding and nesting behaviour.

^ These species are included in the deer-tolerant group as they have been suggested to be positively affected by deer[5,6].

**Supplementary References**


1 Hewson, C. M. & Noble, D. G. Population trends of breeding birds in British woodlands over a 32-year period: relationships with food, habitat use and migratory behaviour. *Ibis* **151**, 464-486 (2009).

2 Vanhinsbergh, D., Fuller, R. J. & Noble, D. An analysis of changes in the populations of British woodland birds and a review of possible causes. (British Trust for Ornithology, 2001).

3 Newson, S. E., Johnston, A., Renwick, A. R., Baillie, S. R. & Fuller, R. J. Modelling large-scale relationships between changes in woodland deer and bird populations. *Journal of Applied Ecology* **49**, 278-286 (2012).

4 Lewis, A. J. G., Amar, A., Cordi-Piec, D. & Thewlis, R. M. Factors influencing Willow Tit Poecile montanus site occupancy: a comparison of abandoned and occupied woods. *Ibis* **149**, 205-213 (2007).

5 Gill, R. M. A. & Fuller, R. J. The effects of deer browsing on woodland structure and songbirds in lowland Britain. *Ibis* **149**, 119-127 (2007).

6 Fuller, R. J. Responses of woodland birds to increasing numbers of deer: a review of evidence and mechanisms. *Forestry* **74**, 289-298 (2001).
